# Ethyl 1-(6-chloro-3-pyridylmeth­yl)-5-methyl-1*H*-1,2,3-triazole-4-carboxyl­ate

**DOI:** 10.1107/S1600536808034430

**Published:** 2008-11-13

**Authors:** Tian-Jia Hua, Feng-Mei Sun, Wen-Ju Liu, Yong-Nian Qu

**Affiliations:** a Department of Medicinal Chemistry, Yunyang Medical College, Shiyan 442000, People’s Republic of China; b School of Chemistry and Chemical Engineering, Henan Institute of Science and Technology, Xinxiang, 453003, People’s Republic of China; c Department of Chemistry and Life Science, Xianning College, Xianning 4371000, People’s Republic of China

## Abstract

In the title compound, C_12_H_13_ClN_4_O_2_, the triazole ring carries methyl and ethoxy­carbonyl groups, and is bound *via* a methyl­ene bridge to a chloro­pyridine unit. There is evidence for significant electron delocalization in the triazolyl system. Intra­molecular C—H⋯O and inter­molecular C—H⋯N hydrogen bonds stabilize the structure.

## Related literature

For applications of triazoles, see: Abu-Orabi *et al.* (1989 ▶); Fan & Katritzky (1996 ▶); Dehne (1994 ▶); Wang *et al.* (1998 ▶). For bond-length data, see: Sasada (1984 ▶).
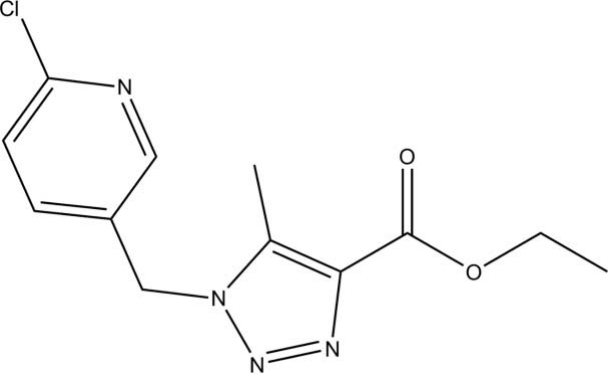


## Experimental

### 

#### Crystal data

C_12_H_13_ClN_4_O_2_
*M* *_r_* = 280.71
Monoclinic, 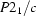

*a* = 24.984 (4) Å
*b* = 4.3919 (8) Å
*c* = 12.040 (2) Å
β = 94.415 (2)°
*V* = 1317.2 (4) Å^3^
*Z* = 4
Mo *K*α radiation
μ = 0.29 mm^−1^
*T* = 291 (2) K
0.46 × 0.38 × 0.33 mm

#### Data collection

Bruker SMART APEX CCD area-detector diffractometer
Absorption correction: none
9219 measured reflections
2450 independent reflections
1991 reflections with *I* > 2σ(*I*)
*R*_int_ = 0.023

#### Refinement

*R*[*F* ^2^ > 2σ(*F* ^2^)] = 0.039
*wR*(*F* ^2^) = 0.105
*S* = 1.05
2450 reflections
174 parameters
H-atom parameters constrained
Δρ_max_ = 0.18 e Å^−3^
Δρ_min_ = −0.23 e Å^−3^

### 

Data collection: *SMART* (Bruker, 2000 ▶); cell refinement: *SAINT* (Bruker, 2000 ▶); data reduction: *SAINT*; program(s) used to solve structure: *SHELXS97* (Sheldrick, 2008 ▶); program(s) used to refine structure: *SHELXL97* (Sheldrick, 2008 ▶); molecular graphics: *SHELXTL* (Sheldrick, 2008 ▶); software used to prepare material for publication: *SHELXTL*.

## Supplementary Material

Crystal structure: contains datablocks global, I. DOI: 10.1107/S1600536808034430/at2655sup1.cif

Structure factors: contains datablocks I. DOI: 10.1107/S1600536808034430/at2655Isup2.hkl

Additional supplementary materials:  crystallographic information; 3D view; checkCIF report

## Figures and Tables

**Table 1:** Hydrogen-bond geometry (Å, °)

*D*—H⋯*A*	*D*—H	H⋯*A*	*D*⋯*A*	*D*—H⋯*A*
C5—H5⋯N3^i^	0.93	2.57	3.479 (3)	164
C9—H9*A*⋯N4^ii^	0.96	2.59	3.523 (3)	164
C9—H9*B*⋯O2	0.96	2.54	3.114 (3)	119

Symmetry codes: (i) 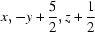; (ii) 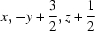.

**Fig. 1.**
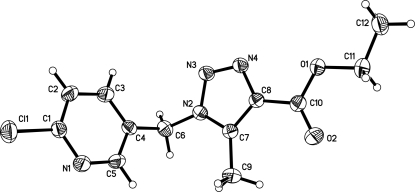
View of the molecular structure of (I), showing the atom labelling schemeand with displacement ellipsoids drawn at the 50% probability level.

**Fig. 2.**
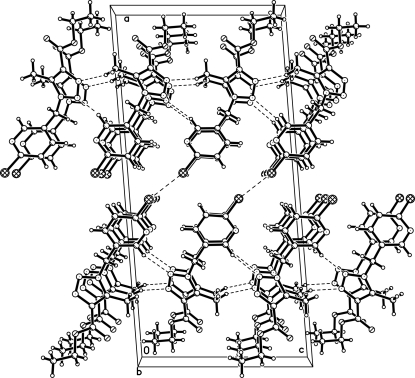
A partial view of the crystal packing of (I), showing the formation of C—H···O and C—H···N hydrogen-bonds (dashed lines).

## supplementary crystallographic information

### Comment

[1,2,3]-Triazoles have been widely used in pharmaceuticals, agrochemicals, dyes, photographic materials, and in corrosion inhibition (Fan & Katritzky, 1996; Dehne,1994; Abu-Orabi *et al.*, 1989). Since the structure-activity relationship is very useful in the rational design of pharmaceuticals and agrochemicals. We report here the crystal structure of the title compound, (I) (Fig. 1), which was synthesized by introducing pyridine rings into a 1,2,3-triazole molecular framework.

The C—N bonds are significantly shorter than a normal single C—N bond [1.47 Å; Sasada, 1984], and closer to the value for a C=N bond [1.28 Å; Wang *et al.*, 1998]. This indicates significant electron delocalization in the triazolyl system.

Intramolecular C—H···O and intermolecular C—H···N hydrogen bonds contribute strongly to the stability of the molecular configuration (Table 1, Fig. 2).

### Experimental

Ethyl acetylacetate (2 mmol) and 5-azidomethyl-2-chloropyridine (2 mmol) were added to a suspension of milled potassium carbonate (2 mmol) in DMSO (10 ml). The mixture was stirred at room temperature for 6 h (monitored by thin-layer chromatography) and poured to water (50 ml). The solid was collected by filtration, washed with water and diethyl ether, respectively, and dried to give 0.52 g of the title compound (yield 91%). Colourless crystals of (I) suitable for X-ray structure analysis were grown from acetone and petroleum ether (2:1, *v*/*v*).

### Refinement

H atoms were placed at calculated positions, with C-H distances of 0.93 (aromatic CH), 0.97 (CH_3_) and 0.97Å (CH_2_). They were refined using a riding model, for methyl H atoms, *U*_iso_(H) = 1.5*U*_eq_(C); for all other H atoms, *U*_iso_(H) = 1.2*U*_eq_(C).

### Crystal data

**Table:**

C_12_H_13_ClN_4_O_2_	*F*_000_ = 584
*M**_r_* = 280.71	*D*_x_ = 1.416 Mg m^−^^3^
Monoclinic, *P*2_1_/*c*	Mo *K*α radiation λ = 0.71073 Å
Hall symbol: -P 2ybc	Cell parameters from 3288 reflections
*a* = 24.984 (4) Å	θ = 3.3–25.5º
*b* = 4.3919 (8) Å	µ = 0.29 mm^−^^1^
*c* = 12.040 (2) Å	*T* = 291 (2) K
β = 94.415 (2)º	Block, colourless
*V* = 1317.2 (4) Å^3^	0.46 × 0.38 × 0.33 mm
*Z* = 4	

### Data collection

**Table:**

Bruker SMART APEX CCD area-detector diffractometer	1991 reflections with *I* > 2σ(*I*)
Radiation source: fine-focus sealed tube	*R*_int_ = 0.023
Monochromator: graphite	θ_max_ = 25.5º
*T* = 291(2) K	θ_min_ = 3.3º
φ and ω scans	*h* = −30→29
Absorption correction: none	*k* = −5→5
9219 measured reflections	*l* = −14→14
2450 independent reflections	

### Refinement

**Table:**

Refinement on *F*^2^	Secondary atom site location: difference Fourier map
Least-squares matrix: full	Hydrogen site location: inferred from neighbouring sites
*R*[*F*^2^ > 2σ(*F*^2^)] = 0.039	H-atom parameters constrained
*wR*(*F*^2^) = 0.105	*w* = 1/[σ^2^(*F*_o_^2^) + (0.0461*P*)^2^ + 0.4886*P*] where *P* = (*F*_o_^2^ + 2*F*_c_^2^)/3
*S* = 1.05	(Δ/σ)_max_ < 0.001
2450 reflections	Δρ_max_ = 0.19 e Å^−^^3^
174 parameters	Δρ_min_ = −0.23 e Å^−^^3^
Primary atom site location: structure-invariant direct methods	Extinction correction: none

### Special details

**Table:**

Geometry. All e.s.d.'s (except the e.s.d. in the dihedral angle between two l.s. planes)are estimated using the full covariance matrix. The cell e.s.d.'s are takeninto account individually in the estimation of e.s.d.'s in distances, anglesand torsion angles; correlations between e.s.d.'s in cell parameters are onlyused when they are defined by crystal symmetry. An approximate (isotropic)treatment of cell e.s.d.'s is used for estimating e.s.d.'s involving l.s. planes.
Refinement. Refinement of *F*^2^ against ALL reflections. The weighted *R*-factor *wR* and goodness of fit *S* are based on *F*^2^, conventional *R*-factors *R* are based on *F*, with *F* set to zero for negative *F*^2^. The threshold expression of *F*^2^ > σ(*F*^2^) is used only for calculating *R*-factors(gt) *etc*. and is not relevant to the choice of reflections for refinement. *R*-factors based on *F*^2^ are statistically about twice as large as those based on *F*, and *R*- factors based on ALL data will be even larger.

### Fractional atomic coordinates and isotropic or equivalent isotropic displacement parameters (Å^2^)

**Table:**

	*x*	*y*	*z*	*U*_iso_*/*U*_eq_	
Cl1	0.46436 (2)	0.66788 (16)	0.65329 (5)	0.0704 (2)	
O1	0.11409 (5)	0.5500 (3)	0.20263 (11)	0.0531 (4)	
O2	0.09737 (6)	0.7643 (4)	0.36514 (13)	0.0737 (5)	
N1	0.37666 (7)	0.9857 (5)	0.61900 (13)	0.0649 (5)	
N2	0.25163 (6)	1.1289 (3)	0.32725 (12)	0.0424 (4)	
N3	0.25643 (6)	0.9861 (4)	0.22765 (12)	0.0489 (4)	
N4	0.21318 (6)	0.8240 (4)	0.20603 (13)	0.0471 (4)	
C1	0.41512 (7)	0.8561 (5)	0.56801 (16)	0.0484 (5)	
C2	0.41911 (8)	0.8610 (6)	0.45476 (17)	0.0593 (6)	
H2	0.4473	0.7647	0.4228	0.071*	
C3	0.37992 (8)	1.0133 (6)	0.39049 (16)	0.0576 (6)	
H3	0.3814	1.0215	0.3136	0.069*	
C4	0.33851 (7)	1.1538 (4)	0.43968 (14)	0.0426 (4)	
C5	0.33924 (9)	1.1340 (6)	0.55394 (16)	0.0625 (6)	
H5	0.3118	1.2305	0.5885	0.075*	
C6	0.29525 (8)	1.3267 (5)	0.37236 (17)	0.0507 (5)	
H6A	0.2806	1.4811	0.4191	0.061*	
H6B	0.3109	1.4293	0.3113	0.061*	
C7	0.20468 (7)	1.0550 (4)	0.36960 (14)	0.0415 (4)	
C8	0.18046 (7)	0.8611 (4)	0.29077 (14)	0.0413 (4)	
C9	0.18798 (9)	1.1716 (5)	0.47790 (16)	0.0597 (6)	
H9A	0.2023	1.0422	0.5372	0.090*	
H9B	0.1495	1.1728	0.4764	0.090*	
H9C	0.2013	1.3748	0.4899	0.090*	
C10	0.12697 (8)	0.7226 (5)	0.29187 (16)	0.0477 (5)	
C11	0.06017 (8)	0.4189 (6)	0.19684 (19)	0.0606 (6)	
H11A	0.0336	0.5791	0.1999	0.073*	
H11B	0.0567	0.2824	0.2592	0.073*	
C12	0.05159 (10)	0.2481 (6)	0.0894 (2)	0.0700 (7)	
H12A	0.0555	0.3845	0.0283	0.105*	
H12B	0.0161	0.1623	0.0833	0.105*	
H12C	0.0776	0.0878	0.0878	0.105*	

### Atomic displacement parameters (Å^2^)

**Table:**

	*U*^11^	*U*^22^	*U*^33^	*U*^12^	*U*^13^	*U*^23^
Cl1	0.0538 (3)	0.0928 (5)	0.0629 (4)	0.0083 (3)	−0.0057 (2)	0.0110 (3)
O1	0.0436 (7)	0.0630 (9)	0.0533 (8)	−0.0077 (6)	0.0074 (6)	−0.0061 (7)
O2	0.0623 (9)	0.1011 (13)	0.0607 (9)	−0.0154 (9)	0.0240 (8)	−0.0149 (9)
N1	0.0567 (10)	0.1005 (15)	0.0377 (9)	0.0146 (10)	0.0050 (8)	−0.0002 (10)
N2	0.0477 (8)	0.0423 (8)	0.0367 (8)	0.0002 (7)	0.0010 (6)	0.0047 (7)
N3	0.0515 (9)	0.0565 (10)	0.0392 (8)	−0.0032 (8)	0.0071 (7)	−0.0004 (7)
N4	0.0486 (9)	0.0538 (10)	0.0391 (8)	−0.0028 (8)	0.0047 (7)	−0.0002 (7)
C1	0.0432 (10)	0.0573 (12)	0.0444 (10)	−0.0048 (9)	0.0015 (8)	0.0010 (9)
C2	0.0496 (11)	0.0821 (16)	0.0476 (11)	0.0122 (11)	0.0130 (9)	−0.0023 (11)
C3	0.0568 (12)	0.0809 (16)	0.0362 (10)	0.0054 (11)	0.0110 (9)	0.0024 (10)
C4	0.0457 (10)	0.0429 (10)	0.0392 (10)	−0.0068 (8)	0.0029 (7)	−0.0024 (8)
C5	0.0570 (12)	0.0909 (17)	0.0404 (11)	0.0193 (12)	0.0078 (9)	−0.0083 (11)
C6	0.0559 (11)	0.0436 (11)	0.0515 (11)	−0.0048 (9)	−0.0017 (9)	0.0015 (9)
C7	0.0480 (10)	0.0422 (10)	0.0342 (9)	0.0067 (8)	0.0022 (7)	0.0066 (8)
C8	0.0448 (10)	0.0443 (10)	0.0349 (9)	0.0038 (8)	0.0034 (7)	0.0052 (8)
C9	0.0690 (13)	0.0692 (15)	0.0416 (11)	0.0010 (12)	0.0092 (9)	−0.0065 (10)
C10	0.0468 (10)	0.0517 (11)	0.0447 (10)	0.0020 (9)	0.0050 (8)	0.0046 (9)
C11	0.0448 (11)	0.0683 (14)	0.0692 (14)	−0.0100 (10)	0.0085 (10)	−0.0011 (11)
C12	0.0587 (13)	0.0816 (17)	0.0690 (15)	−0.0187 (12)	0.0004 (11)	−0.0038 (13)

### Geometric parameters (Å, °)

**Table:**

Cl1—C1	1.748 (2)	C4—C6	1.505 (3)
O1—C10	1.334 (2)	C5—H5	0.9300
O1—C11	1.462 (2)	C6—H6A	0.9700
O2—C10	1.208 (2)	C6—H6B	0.9700
N1—C1	1.310 (3)	C7—C8	1.380 (3)
N1—C5	1.341 (3)	C7—C9	1.490 (3)
N2—C7	1.354 (2)	C8—C10	1.469 (3)
N2—N3	1.367 (2)	C9—H9A	0.9600
N2—C6	1.465 (2)	C9—H9B	0.9600
N3—N4	1.303 (2)	C9—H9C	0.9600
N4—C8	1.365 (2)	C11—C12	1.496 (3)
C1—C2	1.375 (3)	C11—H11A	0.9700
C2—C3	1.374 (3)	C11—H11B	0.9700
C2—H2	0.9300	C12—H12A	0.9600
C3—C4	1.377 (3)	C12—H12B	0.9600
C3—H3	0.9300	C12—H12C	0.9600
C4—C5	1.377 (3)		
			
C10—O1—C11	115.24 (15)	N2—C7—C8	103.62 (15)
C1—N1—C5	116.16 (17)	N2—C7—C9	123.82 (17)
C7—N2—N3	110.96 (15)	C8—C7—C9	132.56 (18)
C7—N2—C6	130.06 (16)	N4—C8—C7	109.36 (16)
N3—N2—C6	118.97 (15)	N4—C8—C10	123.69 (16)
N4—N3—N2	107.36 (14)	C7—C8—C10	126.89 (16)
N3—N4—C8	108.69 (15)	C7—C9—H9A	109.5
N1—C1—C2	124.75 (19)	C7—C9—H9B	109.5
N1—C1—Cl1	116.01 (15)	H9A—C9—H9B	109.5
C2—C1—Cl1	119.23 (16)	C7—C9—H9C	109.5
C3—C2—C1	117.59 (18)	H9A—C9—H9C	109.5
C3—C2—H2	121.2	H9B—C9—H9C	109.5
C1—C2—H2	121.2	O2—C10—O1	123.45 (18)
C2—C3—C4	120.15 (18)	O2—C10—C8	123.52 (19)
C2—C3—H3	119.9	O1—C10—C8	113.02 (16)
C4—C3—H3	119.9	O1—C11—C12	107.99 (17)
C5—C4—C3	116.66 (18)	O1—C11—H11A	110.1
C5—C4—C6	121.56 (17)	C12—C11—H11A	110.1
C3—C4—C6	121.77 (17)	O1—C11—H11B	110.1
N1—C5—C4	124.68 (19)	C12—C11—H11B	110.1
N1—C5—H5	117.7	H11A—C11—H11B	108.4
C4—C5—H5	117.7	C11—C12—H12A	109.5
N2—C6—C4	112.53 (15)	C11—C12—H12B	109.5
N2—C6—H6A	109.1	H12A—C12—H12B	109.5
C4—C6—H6A	109.1	C11—C12—H12C	109.5
N2—C6—H6B	109.1	H12A—C12—H12C	109.5
C4—C6—H6B	109.1	H12B—C12—H12C	109.5
H6A—C6—H6B	107.8		
			
C7—N2—N3—N4	0.3 (2)	N3—N2—C7—C8	−0.43 (19)
C6—N2—N3—N4	179.64 (15)	C6—N2—C7—C8	−179.72 (17)
N2—N3—N4—C8	0.0 (2)	N3—N2—C7—C9	179.32 (17)
C5—N1—C1—C2	−0.5 (3)	C6—N2—C7—C9	0.0 (3)
C5—N1—C1—Cl1	179.07 (18)	N3—N4—C8—C7	−0.3 (2)
N1—C1—C2—C3	0.1 (4)	N3—N4—C8—C10	177.11 (17)
Cl1—C1—C2—C3	−179.46 (17)	N2—C7—C8—N4	0.5 (2)
C1—C2—C3—C4	−0.1 (3)	C9—C7—C8—N4	−179.3 (2)
C2—C3—C4—C5	0.5 (3)	N2—C7—C8—C10	−176.87 (17)
C2—C3—C4—C6	179.1 (2)	C9—C7—C8—C10	3.4 (3)
C1—N1—C5—C4	1.0 (4)	C11—O1—C10—O2	1.9 (3)
C3—C4—C5—N1	−1.0 (4)	C11—O1—C10—C8	−177.09 (17)
C6—C4—C5—N1	−179.6 (2)	N4—C8—C10—O2	−177.4 (2)
C7—N2—C6—C4	93.0 (2)	C7—C8—C10—O2	−0.4 (3)
N3—N2—C6—C4	−86.3 (2)	N4—C8—C10—O1	1.6 (3)
C5—C4—C6—N2	−96.2 (2)	C7—C8—C10—O1	178.57 (17)
C3—C4—C6—N2	85.3 (2)	C10—O1—C11—C12	176.97 (19)

### Hydrogen-bond geometry (Å, °)

**Table:**

*D*—H···*A*	*D*—H	H···*A*	*D*···*A*	*D*—H···*A*
C5—H5···N3^i^	0.93	2.57	3.479 (3)	164
C9—H9A···N4^ii^	0.96	2.59	3.523 (3)	164
C9—H9B···O2	0.96	2.54	3.114 (3)	119

Symmetry codes: (i) *x*, −*y*+5/2, *z*+1/2; (ii) *x*, −*y*+3/2, *z*+1/2.
